# Enzyme Inhibitors: The Best Strategy to Tackle Superbug NDM-1 and Its Variants

**DOI:** 10.3390/ijms23010197

**Published:** 2021-12-24

**Authors:** Xiaoting Li, Dongmei Zhao, Weina Li, Jichao Sun, Xiuying Zhang

**Affiliations:** 1 Heilongjiang Key Laboratory for Animal Disease Control and Pharmaceutical Development, Northeast Agricultural University, Harbin 150036, China; lixting3069@163.com (X.L.); z18103693767@163.com (D.Z.); nb208493@163.com (W.L.); caassjc@163.com (J.S.); 2 Department of Basic Veterinary Science, College of Veterinary Medicine, Northeast Agricultural University, Harbin 150036, China

**Keywords:** NDM-1, multidrug resistance, variants, enzyme inhibitors, pharmacophore, biological activity

## Abstract

Multidrug bacterial resistance endangers clinically effective antimicrobial therapy and continues to cause major public health problems, which have been upgraded to unprecedented levels in recent years, worldwide. β-Lactam antibiotics have become an important weapon to fight against pathogen infections due to their broad spectrum. Unfortunately, the emergence of antibiotic resistance genes (ARGs) has severely astricted the application of β-lactam antibiotics. Of these, New Delhi metallo-β-lactamase-1 (NDM-1) represents the most disturbing development due to its substrate promiscuity, the appearance of variants, and transferability. Given the clinical correlation of β-lactam antibiotics and NDM-1-mediated resistance, the discovery, and development of combination drugs, including NDM-1 inhibitors, for NDM-1 bacterial infections, seems particularly attractive and urgent. This review summarizes the research related to the development and optimization of effective NDM-1 inhibitors. The detailed generalization of crystal structure, enzyme activity center and catalytic mechanism, variants and global distribution, mechanism of action of existing inhibitors, and the development of scaffolds provides a reference for finding potential clinically effective NDM-1 inhibitors against drug-resistant bacteria.

## 1. Introduction

Abusing the use of antibiotics causes the mass production of resistant bacteria and resistance genes. Resistant bacteria and resistance genes can be transmitted to humans via the food chain and environments, which leads to a variety of infectious illnesses, including norovirus and hepatitis A [1,2,3]. The emergence and spread of multidrug-resistant bacteria (MDR) and antibiotic resistance genes (ARGs) have become an international public health crisis [4,5]. A large number of studies have shown that MDR mainly focuses on gram-negative bacteria, especially carbapenem-resistant *Enterobacteriaceae* (CRE) and carbapenem-resistant *Acinetobacter baumannii* (CRAB) [6,7]. With the increasing awareness of MDR and ARGs in the world, proposing strategies to prevent and control clinical infection is an urgent task. β-Lactam antibiotics are the most widely used antibacterial agents at present and block bacterial cell walls due to their covalent bond with basic penicillin-binding proteins (PBPs) [8]. Each species of bacteria has its own unique set of PBPs, and each species of bacteria can have 3 to 8 enzymes [9]. However, bacteria have developed sophisticated resistance mechanisms to resist treatments with β-lactam antibiotics. Among them, β-lactamases (BLs) represent the most extensive and clinically relevant mechanism [10,11]. BLs are expressed by both gram-positive and gram-negative bacteria that hydrolyze the β-lactam ring, resulting in the inactivation of the drug [12].

The Ambler scheme classifies BLs into four classes according to the protein homology of enzymes [13]. Classes A, C, and D are serine β-lactamases (SBLs) (such as AmpC, KPC, OXA), which can catalyze the hydrolysis of β-lactam antibiotics by nucleophile serine, and a transient covalent reaction occurs at the active sites [14,15,16]. Class B BLs, also known as metallo β-lactamases (MBLs) (such as IMP, VIM, NDM), catalyze the hydrolysis of β-lactam antibiotics through a non-covalent mechanism and are characterized by one or two equivalents of bound zinc (Zn) ions that are indispensable for enzyme activity [17,18]. MBLs are further divided into three subclasses (B1, B2, and B3), which are mainly defined by differences in the primary zinc coordination layer [19]. New Delhi metallo-β-lactamase-1 (NDM-1) [20,21] is a new member of the B1 subclass of the MBL superfamily and was first identified in 2008 by *Klebsiella pneumoniae* (*K. pneumoniae*) isolated from a Swedish patient [22,23,24]. Subsequently, NDM-1 has been characterized mainly in *Escherichia coli* (*E. coli*), *Acinetobacter* spp., and (*K. pneumoniae*) [25,26]. The global appearance of NDM-1 can effectively hydrolyze almost all available β-lactam antibiotics except for monobactams such as aztreonam, and it spreads quickly between the same species or even different species [27]. Plasmids mediate the *bla*NDM-1 gene transfer among the same and even different bacterial species, endangering efficacious antibacterial treatments [28,29]. Moreover, NDM-1 positive bacteria have been detected in drinking water and wastewater [30]. Only colistin and tigecycline are effective against NDM-1 producing bacteria, and some NDM-1 producing bacteria are also resistant to these two drugs [31,32]. Ongoing research has suggested that new types of antibiotics will have long research and development periods and high costs. At present, the most effective method against NDM-1 is to design an inhibitor to protect β-lactam drugs from enzyme hydrolysis. In addition, combining β-lactam drugs with inhibitors can restore its antibacterial activity and kill pathogenic microorganisms. Although the structural features and information about the mechanism of NDM-1 hydrolysis have been evaluated based on its homologs, it is still a challenge to develop an effective NDM-1 inhibitor for treatment. Of course, only a profound understanding of the structure of NDM-1 and the different mechanisms of action of NDM-1 on different substrate types are helpful for the future drug discovery of NDM-1-producing drug-resistant bacteria to avert catastrophic pandemics.

In this review, the major advances in NDM-1 inhibitor discovery and development were presented. This summary focuses on the effective structural basis and inhibitory mechanism of inhibitors and inhibitors that synergize with β-lactam antibiotics to restore the drug sensitivity of clinically relevant NDM-1-expressing bacteria in vitro and in vivo. 

## 2. The Structure of NDM-1

**Figure 1:**
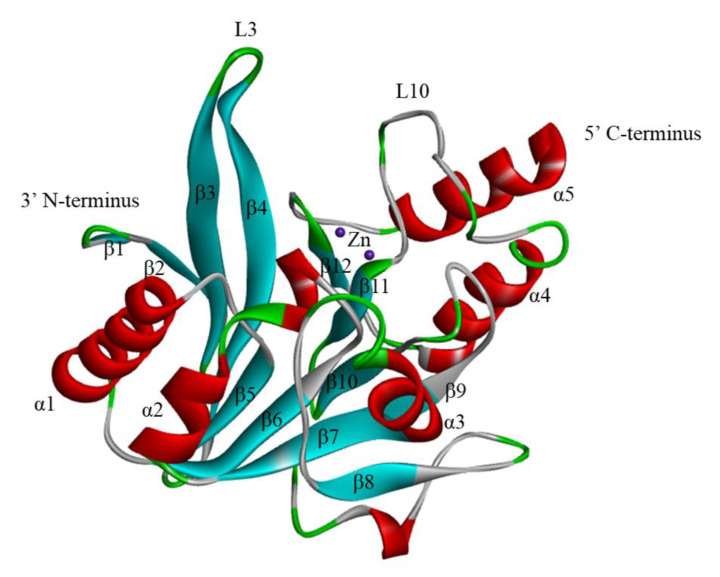
The Crystal Structure of NDM-1 (PDB: 3SPU). The two zinc ions are shown as purple spheres. α-Helices and β-strands are colored red and blue, respectively. The secondary structure designations are labeled.

The NDM-1-encoding gene *bla*NDM-1 is mostly located on readily transferable plasmids, and NDM-1 consists of 270 amino acids and is expressed at approximately 27.5 kDa [33,34]. NDM-1 is a single-chain polypeptide with an N-terminal signal peptide that shuts through the periplasmic space, effectively acts on β-lactam antibiotics, and inactivates the hydrolysis of antibiotics. The NDM-1 enzyme has a compact spherical structure with a size of 50 Å × 40 Å × 40 Å and displays a conical αβ/βα sandwich fold. The αβ/βα fold is unique to the MBL superfamily with two central antiparallel β-sheets flanked by two pairs of α-helices. The hydrolysis mechanism of the substrate catalyzed by BLs indicates that the NDM-1 enzyme folded spatial structure is highly adaptable to β-lactam antibiotics. The right portion (C-terminal) of the NDM-1 molecule consists of two α-helices (α4 and α5) and five antiparallel β-strands (β8–β12). The left subdomain (N-terminal) consists of two α-helices (α1 and α2) and seven antiparallel β-strands (β1–β7) (Figure 1) [12,35,36]. The β-chain interacts through hydrophobic groups, the N-terminal β-chain is highly twisted with a twist angle > 100 degrees, and α-helices and β-strands are connected by a flexible loop [37].

In solution, the active form of NDM-1 is reported as a monomer, which is similar to other B1 MBLs. In some cases, due to hydrophobic and van der Waals interactions, NDM-1 may exist in the form of a partial dimer, and the loop insertion sequence (Thr162–Gly167) is considered to contribute to the dimerization of NDM-1 [38,39]. It is speculated that NDM-1 can exist as a dimer in both membrane-bound and soluble states, which contributes to the formation of the unique mechanism of resistance of NDM-1 [40].

**Figure 2:**
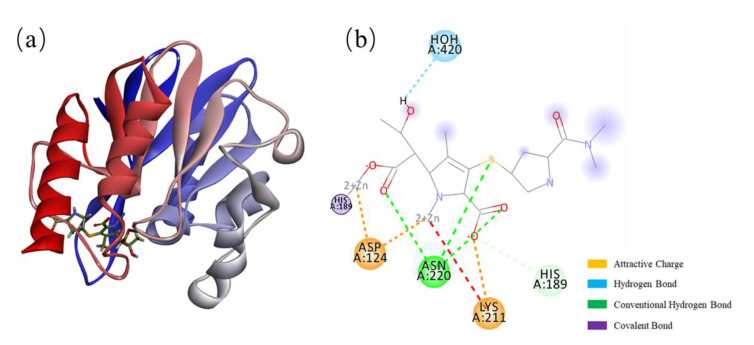
Crystal structure of NDM-1 bound to hydrolyzed meropenem (PDB: 4EYL). (**a**) Structure of the docking complex of NDM-1 with meropenem. (**b**) A ligand interaction diagram for NDM-1 and meropenem in the active site.

Many studies are describing the characteristics of NDM-1. To date, many reports of NDM-1 crystal structural determinations have been deposited in the Protein Data Bank (PDB) [41]. These structural models showed that NDM-1 has a typical MBL fold, but a considerable degree of variation exists among the models. These differences include the cocrystallized substrate, the stoichiometry of the bound metal ion, and the conformation of the ligand with substrate binding loops [39]. Dozens of crystal structures of NDM-1 cocrystallized with hydrolyzed antibiotics, including ampicillin (5ZGQ), benzylpenicillin (4EYF), methicillin (4EY2), oxacillin (4EYB), and meropenem (4EYL) (Figure 2) [42,43]. There are also crystal structures of NDM-1 combined with potential inhibitors, such as L-captopril (4EXS) and bisthiazolidine (4U4L). Comparing the root-mean-square deviation (RMSD) values of several different NDM-1 crystal structures, it was found that Loop 3 (residues 63 to 73) contained the greatest flexibility, and the other regions of the protein skeleton were relatively stable [44,45]. The crystal structure of NDM-1 cocrystallized with hydrolyzed antibiotics, which reveals the structure-activity relationship (SAR) between the NDM-1 enzyme and its substrate, provides the basis for exploring the mechanism of hydrolyzing antibiotics and broad substrate specificity which further deepens our understanding of these important enzymes.

## 3. Active Site and Hydrolysis Mechanism of NDM-1

**Figure 3:**
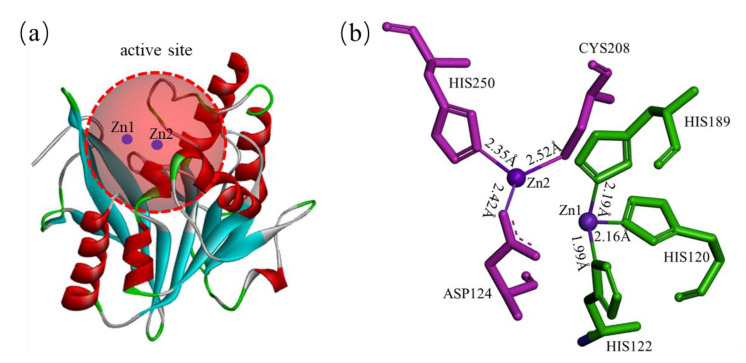
The structure of the NDM-1 active site. (**a**) The red circle indicates the active site region of NDM-1. (**b**) Coordination distance between the key amino acids of NDM-1 and zinc.

In the active site of NDM-1, there are two divalent zinc ions connected by hydroxide ions that interact with different amino acid residues [46,47]. Zn1 is tetrahedrally coordinated with three histidine residues, His120, His122, and His189, with distances of 2.16 Å, 1.99 Å, and 2.19 Å, respectively. Zn2 coordinates with Asp124, Cys208, and His250 at distances of 2.42 Å, 2.52 Å, and 2.35 Å, respectively [35,42,48] (Figure 3). In addition, Zn2 coordinates with three water molecules, one of which acts as a catalytic water molecule and interacts with Zn1 and Zn2. The catalytic water molecule is most likely in the form of hydroxide ions, which form a bridge between the two zinc ions and act as nucleophiles during enzymatic substrate hydrolysis [49,50,51,52]. It is assumed that the position of nucleophilic hydroxide is similar to that of all β-lactam antibiotic product complexes, and it is directly located between Zn 1 and Zn 2, with distances of 2.0 ± 0.1 Å and 3.0 ± 0.1 Å, respectively [53].

**Figure 4:**
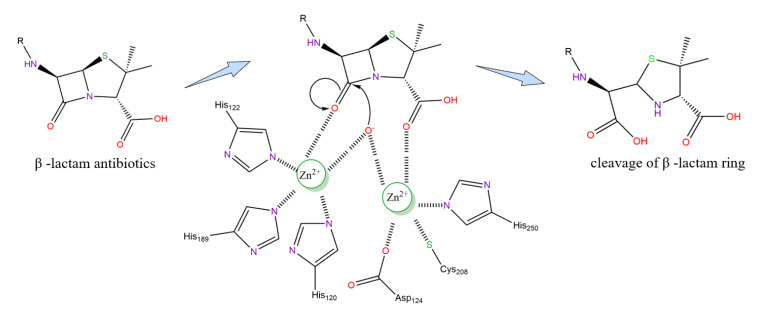
Proposed mechanism hydrolytic mechanism of NDM-1.

In the current proposed NDM-1 enzyme structure model, two zinc ions with a distance of 3.2 Å are connected by the side chain of Asp124 [36,53]. Therefore, Zn-Zn ions bind closely during the formation of substrate and enzyme complexes and promote the interaction of Zn2 with the amide groups of the β-lactam ring [54]. In addition, the distance of Zn1-Zn2 in the substrate structure increase after hydrolysis, thereby weakening the interaction and releasing the hydrolysate from the NDM-1 enzyme activity center [55]. Zn1 keeps the hydroxyl group in the correct direction to attack the carbon atom on the carbonyl group of the β-lactam ring by nucleophilicity, while the oxygen atom of the carboxyl group is positioned in a way that enables it to interact with the Zn2 ion [56,57]. The research also shows that the hydroxide ion at the active site attacks the carbonyl carbon of β-lactam to form an intermediate, and the intermediate is stabilized by zinc ions, thus forming a transition state complex and finally leading to the cleavage of the C-N bond (Figure 4). The catalytic mechanism of NDM-1 is still unclear, and some researchers believe that it follows the dual-zinc catalytic mechanism described above. By only thoroughly understanding the relationship between the active site and the mechanism of hydrolysis, a potential inhibitor may be designed as a future treatment, which is needed in the present situation [58,59].

## 4. NDM-1 Variants and Global Distribution

**Figure 5:**
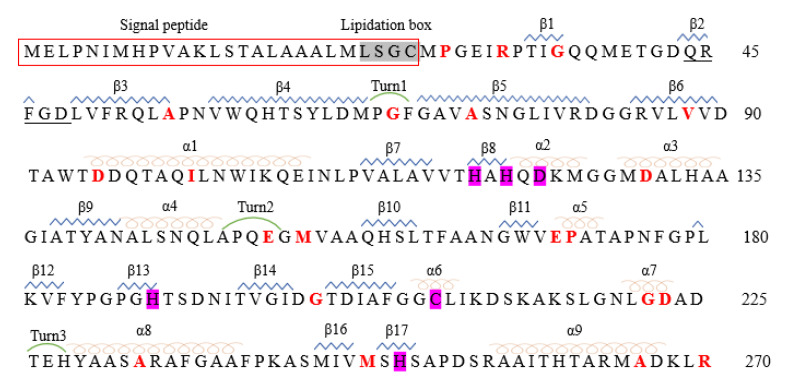
NDM-1 amino acid sequence and NDM-1 point mutations. The annotation of the NDM amino acid sequence was adopted from data reported under UniPort. Signal peptides of NDM-1 are framed with red lines. α-helices, β-strands, and turns are indicated as orange spirals and blue zigzag lines and green apsidal lines, respectively. The zinc-binding residues are highlighted in purple. The lipidation box is highlighted in gray.

The selective pressure caused by the increasing use and abuse of broad-spectrum antibiotics is considered to play a key role in the evolution of NDM-1, leading to the emergence of NDM variants [60,61]. NDM variants are characterized by point mutations at specific positions. To date, substitutions at 21 different positions of the 270 amino acids have been reported, which resulted in 31 different NDM mutations [61,62,63] (Figure 5). Among 31 known NDM variants, the amino acid substitution pattern is usually between 1 and 5. NDM-2, -3, -4, -6, -9, -11, -14, -22, -23, -24, -25, -28, -29, -30 and -31 are different from NDM-1 in that they are replaced by a single amino acid, while the rest of them are different due to multiple substitutions. One exception is NDM-18, which is identical to NDM-1, and the only difference is the 5-amino acid tandem repeat sequence (QRFGD) at positions 44–48 of NDM-1 [64]. The secondary structure and thermal stability of the NDM-1 variant were detected by circular dichroism (CD) and differential scanning fluorescence (DSF), and no amino acid mutation was found to affect the whole folding of the NDM-1 variants [65]. However, substitution with different amino acid residues either in various combinations or individually has different effects on the catalytic activity and thermal stabilities of the enzyme in comparison to NDM-1 [66,67,68].

In the process of NDM evolution under the selection of environmental pressure, residue M154 appears to be the most frequently replaced, among which the mutation of M154L is the most common change (observed in 14 of the 31 distinct NDM variants) and M154V occurred once (in NDM-11). The mutation M154L, which appears on the surface of the protein together with other mutations, is regarded as one of the most favorable substitutions, as it enhances the metal-binding affinity and may improve thermostability through other mechanisms, such as eliminating a charge in one of two closely situated aspartate residues (D130N and D95N) or reducing the flexibility of the loop (G222D) [61,69,70]. Several mutations may introduce other mutations as global suppressors to stabilize the hydrophobic core of NDM, especially M154L, which leads to additional function at the cost of structural instability [71]. Global-suppressing mutations have been researched in the evolution of SBLs [72].

To date, it has been observed that amino acid substitutions in NDM variants are far from the key catalytic residues or those residues related to maintaining active site conformation or substrate interaction. However, some variants have been reported to change the activities against β -lactam [73], which suggests that a substitution, despite being located at a nonactive site, may distort the groove, resulting in a major impact on enzyme activity, and the mechanism of the increase in activity remains unclear [74]. Observing the distribution of all substitutions in the crystal structure of NDM-1 mutants, it was found that the loop regions were the most susceptible to mutations. More than half of the substitutions are located within the loops of the NDM structure, followed by the α-helices and then in the β-strands (Table 1). The loop region is also mainly involved in mediating drug resistance, which indicates that substitutions in the loops may provide an evolutionary advantage for bacteria [75,76].

It is speculated that various amino acid substitutions affect the stability and catalytic activity of the enzyme. NDM variants containing the V88L substitution of NDM-5, -16, -17, -21, and -24 have been reported to show higher carbapenem activity than that of NDM-1, while NDM-20 decreased the activity of carbapenems. The minimal inhibitory concentration (MIC) of ertapenem against strains producing NDM-5 -16 and -24 is 4- to 8-fold higher than that of strains producing NDM-1, while strains producing NDM-21 have carbapenem similar to that of NDM-5 [77,78,79,80,81]. Compared with NDM-5 (V88L and M154L), NDM-20 (V88L, M154L, and R270H) improves the hydrolysis activity of some penicillins and cephalosporins but inhibits the activity of carbapenems, which may influence drug strategies in CRE infection [82]. Experimental studies have shown that the affinity of NDM-17 (V88L, M154L, and E170K) for all β-lactams is significantly higher than that of NDM-5 (V88L and M154L), and the increase in its catalytic activity may be related to the novel substitution of E170K. Other substitutions of NDM-4 (M154L) and NDM-14 (D130G) have also been found to increase carbapenem activity [83,84]. However, NDM-8 contains both M154L and D130G substitutions, but enzymatic activities against carbapenems were similar to those of NDM-1 [85]. Regarding NDM-7 (D130N and M154L), in which the Asp at position 130 replaces Asn in NDM-8, it has been reported that the carbapenem activity of NDM-7 is higher than that of NDM-1 [86]. This indicates that the substitution of different amino acid residues on the same site will have different effects on the catalytic activity of the enzyme, which depends on the structure and properties of different amino acids. Above all, these results explained that different combinations of amino acid mutations have different effects on the catalytic activity of NDM-1. In the single substitution variants, it is easy to associate the substitution with an increase or decrease in catalytic activity. However, it is difficult to accurately predict the individual contribution of each substitution in multiple substitution variants. Further comparative kinetic studies can provide insight into the effects of individual substitutes [76,87,88].

NDM variants increase their stability or Zn binding affinity through the accumulation of mutations, while Zn deprivation strictly limits the evolution of this MBL [69]. Under conditions of zinc starvation, NDM-3 (D95N), -4 (M154L), -6 (A233V), -9 (E152k), and -14 (D130G) variants enhance cefotaxime resistance by increasing the metal affinity or stability of NDM enzymes, but the mechanism is still unclear. In contrast, NDM-2(P28A) and NDM -11(M154V) had no significant influence on NDM function under zinc-restricting conditions. NDM-19 (D130N, M154L, and A233V) is a derivative of NDM-7 (D130N and M154L). Under zinc-restricted growth conditions, compared to NDM-1 or NDM-7, NDM-19 shown is less sensitive to carbapenems and cephalosporins [89]. Therefore, the stress caused by zinc depletion may be the major driving force for the evolution of NDM enzymes [61,69]. To date, there has been no research report on new variants of NDM-28, -29, -30, and -31 enzyme activities. Therefore, it is essential to analyze the catalytic efficiency and resistance of evolving NDM variants in the control of clinical infections, which is still an important task.

According to reports, NDM-1 and its variants are spreading worldwide [21,90,91]. The Asian continent is the main storage area for NDM producers, where approximately 58.15% of the abundance of NDM-1 and its variants are distributed, especially in China, Bangladesh, and India [92,93,94]. NDM-2 was the first variant of NDM-1, which spread NDM carbapenemases in A. baumannii, and the corresponding gene mainly spread in the Middle East [95]. However, NDM-1 and its variant total producers in European countries were approximately 16.8%, Romania, Germany, London, etc. with the maximum spread of NDM-1 variant [94,96,97]. NDM-4, -5, and-7 are reported to be prevalent in European subcontinent countries in Italy, Denmark, and France [94,98]. Africa and the American continent account for approximately 10.8% of the global NDM-1-producing countries, of which the Algeria subcontinent and Brazil are the main distribution areas, and NDM-5 is also reported to be distributed in Algeria [99]. Australia accounts for 1.6% of the total NDM-1 producers in the world [100]. All 31 NDM variants detected the highest prevalence in *E. coli* and *K. pneumoniae* species.

## 5. NDM-1 Inhibitors: Discovery and Advances

Although a variety of mechanism-based inhibitors are available for SBLs in therapy, there is still a lack of specific and effective inhibitors against NDM-1 in clinical practice [101,102,103]. The development of effective inhibitors of NDM-1 is impaired by factors such as variability in the entry loop permutation of the active site, the lack of new scaffolds that can selectively target the active site of NDM-1, and the existence of multiple variants of NDM-1 [104,105,106].

The widespread emergence of NDM-1 and its variants has promoted research on antibacterial drugs worldwide. The design and research strategies of drugs targeting NDM-1 have focused on two approaches. First, NDM-1 is combined with carbapenem antibiotics (such as imipenem), which can not only protect the structure of β-lactam antibiotics from being destroyed but also synergize with carbapenem drugs to restore their curative effect on NDM-1. Theoretically, these inhibitors should be β-lactam antibiotics [107]. The second is to design new antibiotics that are insensitive to the catalytic hydrolysis of NDM-1. There is still much progress necessary for new antibiotics that require more effort to innovate and must be explored from scratch. Therefore, most efforts have focused on the first approach, as it offers the ability to protect β-lactam antibiotics from hydrolysis when they are used in combination with inhibitors [108]. Due to the high structural similarity between the MBL subtypes, it is assumed that these inhibitors play a broad role in the MBLs [103].

To date, more than 500 small-molecule compounds have been reported in the literature as potential NDM-1 inhibitors, but there are no clinically approved NDM-1 inhibitors [27]. In this paper, the active chemical scaffolds of NDM-1 inhibitors that have been discovered are reviewed to summarize the pharmacophore and lay a foundation for the subsequent development of NDM-1 inhibitors. NDM-1 inhibitors can be classified and separated into noncovalent inhibitors and covalent inhibitors according to different modes of action.

### 5.1. Noncovalently-Bound Inhibitors

Noncovalent inhibitors mainly consist of zinc-binding inhibitors, boronic acid derivatives, and metal chelating inhibitors.

#### 5.1.1. Zinc-Binding Inhibitors

The reaction mechanism of NDM-1 was investigated through the formation of heterobimetallic analogs (CoCo-, ZnCo-, and CoCd-) and the use of chromatic as a chromogenic substrate. The results show that the zinc ion of NDM-1 is essential in the hydrolysis of β-lactam antibiotics [56]. The electrophilic zinc ions in the NDM-1 enzyme and the electron-rich substituent of NDM-1 inhibitor act synergistically to produce inhibitory activity through ion-dipole interactions.

##### Thiol-Based Derivatives

**Figure 6:**
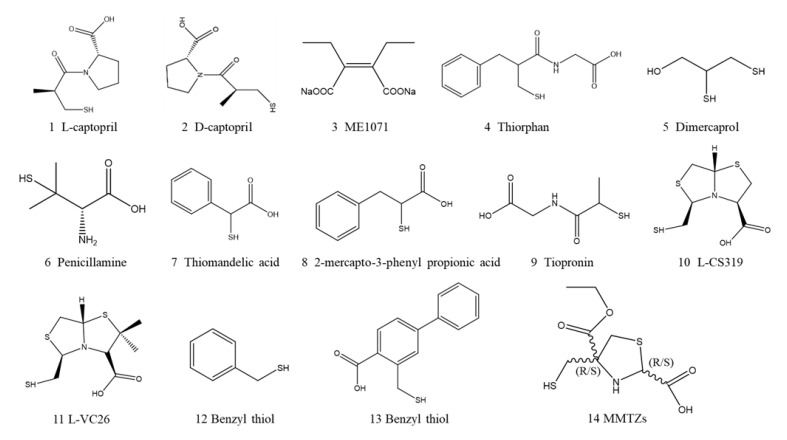
Structures of NDM-1 inhibitors containing thiol groups.

L-captopril (1, Figure 6) is the first angiotensin-converting enzyme (ACE) inhibitor to be clinically approved for the treatment of hypertension [109]. Like ACE, MBLs also contains two key zinc atoms in the active site, so it is no surprise that both enantiomers of captopril are studied as potential NDM-1 inhibitors [110]. D-Captopril (2, Figure 6) has a high inhibitory effect on NDM-1, with a half-maximal inhibitory concentration (IC50) of 7.9 µM, while the inhibitory activity of L-captopril is 25-fold less potent, with an IC50 of 202.0 μM [36]. The crystal structure of NDM-1 combined with L-captopril was obtained by mass spectrometry and X-ray crystallography. The crystal structure showed that the thiol unit of L-captopril was intercalated between Zn1 and Zn2 in the active site of NDM-1, replacing water molecules and becoming a competitive inhibitor of NDM-1 [42]. In addition, Asn220 is a residue involved in intermediate product stabilization and substrate binding. It forms a hydrogen bond with the hydrophilic part of the inhibitor, while the hydrophobic part of the inhibitor interacts with the L3 loop.

Captopril contains thiol and carboxylate pharmacophores, which can form coordination bonds with Zn ions. These chemical groups are the reasons why L- and D-captopril have strong inhibitory effects on all subclasses of MBLs, and these effects have been verified for a long time [111,112,113]. When the carboxylate group of mercaptocarboxylic acids was substituted by bioisosteric groups such as phosphonic acids, the biological activity, and inhibitor binding were reduced, which further demonstrated the importance of the carboxylate acid group [114]. The maleic acid derivative disodium 2,3-diethylmaleate (ME1071, 3 Figure 6) containing carboxylate showed that the activity of carbapenems and cephalosporins on clinical isolates producing MBL was enhanced in vitro [108]. In vivo, the combined application of ME1071 and biapenem can improve the survival rate of ventilator-associated pneumonia in a mouse model caused by MBL-producing *Pseudomonas aeruginosa* [115].

Captopril has been identified as an effective inhibitor of NDM-1, which is the starting point for the development of new inhibitors with thiol and carboxyl pharmacophores. Captopril consists of two units: a 3-mercapto-2-methylpropanoyl fragment and a proline residue. To test various D-captopril analogs produced by substituting proline residues or partially modifying 3-mercapto-2-methylpropanoyl, Klingler and coworkers created a promising platform for screening and developing new MBL inhibitors [116]. This approach has succeeded in finding three FDA-approved drugs possessing a sulfhydryl moiety with a low μM IC 50 range for MBLs. Thiorphan (4, Figure 6), an enkephalin inhibitor, showed high inhibitory activity against NDM-1 with an IC50 of 1.8 μM. Dimercaprol (5, Figure 6) is clinically used as an antidote for metal poisoning and has the lowest IC50 (1.3 μM), and is the most potent. Penicillamine (6, Figure 6) is a drug for Wilson’s disease that also shows NDM-1 enzyme inhibitory activity. Among the thiol-based compounds, thiomandelic acid (7, Figure 6) and 2-mercapto-3-phenyl propionic acid (8, Figure 6) were found to efficiently restore sensitivity to meropenem [117]. The above analogs can restore the imipenem susceptibility of *E. coli* carrying such MBLs. In contrast, tiopronin (9, Figure 6), with an IC50 of 84 μM, has only moderate activity, while N-acetylcysteine has no activity at all [116].

González and coworkers designed and synthesized a novel chemical scaffold bisthiazolidine (BZTS), derivative according to the mechanism of NDM-1 identifying and hydrolyzing β-lactam antibiotics. The BTZ scaffold is a structure with the characteristics of a β-lactam substrate and can be modified by introducing metal-binding groups to target the MBL active site. The modification ability is based on the BTZs bicyclic ring, which could simulate the unbreakable β-lactam ring, retain the bridging nitrogen and the carboxylate that interact with Zn2, and accommodate other metal-binding groups inserted into the necessary Zn center. Four BTZs derivates (L-CS319, L-VC26, and their enantiomers, 10, 11, Figure 6) that behave as competitive NDM-1 inhibitors in vitro, with Ki values in the low micromolar range from 7 to 19 μM, and could restore the efficacy of carbapenem on clinical isolates that produced NDM-1. The two key pharmacophores of the four BTZ derivatives are carboxylate, which interacts with Lys224, and the thiol group and binds to the two Zn ions in the active site of NDM-1 [45]. Therefore, Lys224 was demonstrated to participate in the substrate to identify and adjust substrates toward easy hydrolysis [35,38]. The crystal structure of the most effective L-CS319 and NDM-1 complex (PDB 4u4l) has been clarified, which provides a reference for structural determinants for inhibitor binding and further improves its efficiency [45]. Although compared with NDM-1, other MBL subclasses have different binding modes with BTZs, BTZ scaffolds have also been confirmed to have a broad spectrum of inhibiting all MBL subclasses [118]. This is an effective new strategy to inhibit NDM-1 and provides a valuable scaffold for discovering NDM-1 inhibitors in the future.

In molecular design based on silicon fragments, Cain R and colleagues reported thiol-mediated potent MBL inhibitors [119]. Utilizing a molecular fragment docking approach, benzyl thiol (12, Figure 6) was discovered as a potential competitive inhibitor of NDM-1 with a low micromolar affinity. Docking calculations and polarizable molecular mechanics predicted interactions between the benzyl thiol derivative (13, Figure 6) and NDM-1 [120,121]. The carboxylate forms hydrogen bond interactions with Lys224 and Zn2, the thiol coordinated Zn1 and Zn2, and the aryl ring interact with Trp87 through π-stacking. Nuclear magnetic resonance (NMR) and crystallographic analyses showed that benzyl thiol derivatives were directly combined with MBL active sites. SAR studies have shown that the terminal phenyl ring of derivative modification with heterocycles or substituents revealed has no significant influence on the effectiveness of the designed inhibitor. Within the detection range, none of the individually tested derivatives had any antibacterial activity, but at an active ingredient concentration of 100 µg/mL, the MIC of meropenem to NDM-1-producing strains was decreased. This design can effectively identify the chemotypes of other inhibitors that involve substitution of metal coordination, as well as inhibitors that are dependent on metal coordination.

More recently, 2-mercaptomethyl thiazolidines (MMTZs, 14, Figure 6) were demonstrated to be all MBL inhibitors; they contain a thiazolidine ring and two chiral carbon centers with free thiol groups and carboxylates. MMTZs inhibit MBLs, including NDM-1, by maintaining a conserved binding mode, which utilizes thiol-coordinated mono- or dizinc centers, and thiazolidine sulfur interact with aromatic residue f active site. In vitro, MMTZs inhibit all MBL subclasses with Ki values ranging from 0.16 μM to 130 μM and can restore the activity of carbapenems against NDM-1-expressing recombinant E. coli. Therefore, MMTZs represent a promising MBL inhibitor scaffold to control the emergence of bacterial resistance [122].

##### Rhodanine

**Figure 7:**
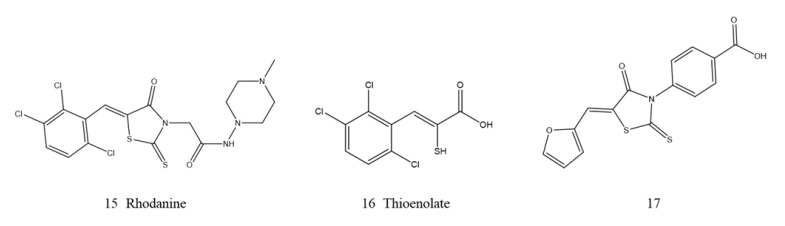
Rhodanine derivatives as NDM-1 inhibitors.

Rhodanine (15, Figure 7) is one of the few compounds with inhibitory activity in all enzyme classes. Due to the noncompetitive or competitive inhibition of PBPs, some rhodanines also have antibacterial activity [123,124]. Later, Brem et al., through crystallographic analyses of the mechanism by which rhodanine inhibits VIM-2 MBL, revealed that the rhodanine ring was hydrolyzed into thioenolate (16, Figure 7), and thioenolate was bound by dizinc chelation. Crystallographic observations and NMR analyses in solution revealed that the thioenolate derived from rhodanine as an efficient broad-spectrum MBL inhibitor [125]. Synthesis and characterization of 26 rhodanine derivatives and 1 thioenolate derivative to develop broad-spectrum MBL inhibitors. The biochemical evaluation revealed that most of the tested derivatives strongly inhibited B3 MBL. In particular, for NDM-1, compound 17 (17, Figure 7) was the most active. SAR and Dock showed that the substitution of rhodanine with diaryl provides a good scaffold for the design of broad-spectrum inhibitors of MβLs [126].

##### Thiadiazoles

**Figure 8:**
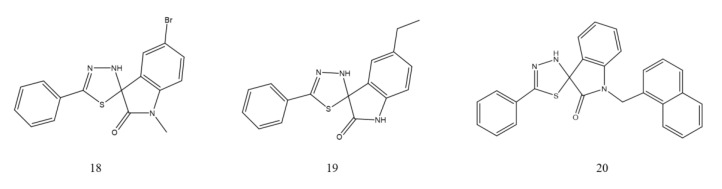
Thiadiazoles derivatives as NDM-1 inhibitors.

A high-throughput screening method was established by Falconer B and coworkers to discover the chemical perturbants of the essential. Therefore, two spiro-indoline-thiadiazoles (5-ethyl-5′-phenyl-3′H-spiro[indoline-3,2′-[1,3,4] thiadiazol]-2 and 5-bromo-1-methyl-5′-phenyl-3′H-spiro[indoline-3,2′-[1,3,4] thiadiazol]-2) (18, 19, Figure 8) that disrupt iron homeostasis in bacteria have been identified and characterized. These two compounds are intracellular chelators with two isomeric states. Various spiro-heterocyclic compounds were converted into the open chelating form of merocyanine, thus enhancing the antibacterial activity of β-lactams [127]. Then, researchers also verified that a range of spiro-indoline-thiadiazole analogs would potentiate β-lactam antibiotics on NDM-1-positive *K. pneumoniae* in vitro by resisting zinc availability. Among analogs, compound 20 (20, Figure 8) inhibited NDM-1 in vitro and combined with meropenem, the bacterial load in the liver and spleen of mouse peritonea infected by NDM-1-expressing *K. pneumoniae* was significantly reduced [128].

##### Isatin-β-thiosemicarbazones (IBT)

**Figure 9:**
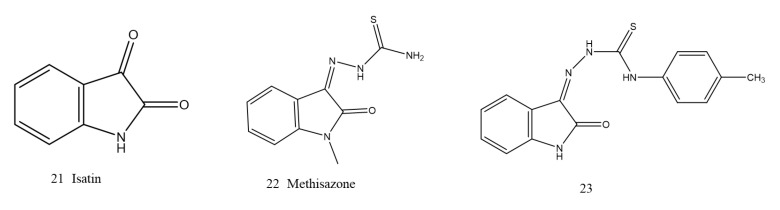
Isatin-β-thiosemicarbazones derivatives as NDM-1 inhibitors.

Repurposing old drugs is the most effective foundational method for the discovery of new drugs [129]. Isatin (21, Figure 9) is an endogenous natural product, and its derivatives possess antibacterial, antifungal, antimalarial and antiviral biological activities [130]. It was found that methisazone (22, Figure 9) was a weak NDM-1 inhibitor with an IC50 of 297.6 µM. Docking studies have shown that the thiol group present on the molecule forms several coordination bonds with zinc ions, indicating that thiol groups play an important role in the inhibition process. Inspired by this result, a series of IBT derivatives sharing the methisazone scaffold were designed and evaluated as new NDM-1 inhibitors. The IC50 values of the nine IBT derivatives were all below 10 μM, among which compound 23 (23, Figure 9) was the strongest at 2.72 µmol/L [131]. When new compounds were screened against drug-resistant bacteria, a few IBTs that can inhibit the growth of MRSA and VRE were also discovered, and their derivatives had significant activity against gram-positive bacteria. This finding indicated that IBTs can be regarded as potential lead compounds for discovering NDM-1 inhibitors [132].

##### Pyridine Dicarboxylic Acid Derivatives

**Figure 10:**
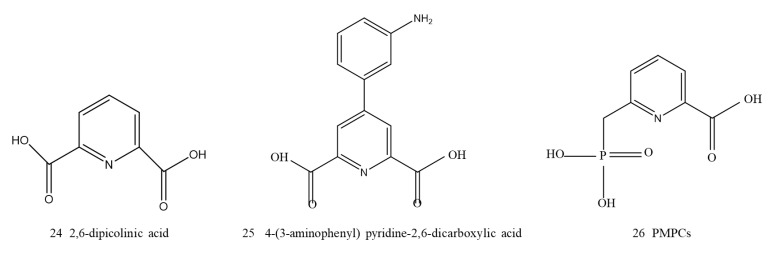
Pyridine dicarboxylic acid derivatives as NDM-1 inhibitors.

Chen and coworkers used a fragment-based NDM-1 inhibitor discovery strategy. 2,6-Dipicolinic acid (DPA, 24, Figure 10) was identified as a valuable chemical scaffold with an IC50 of 0.52 μM and can be used to develop a novel type of broad-spectrum MBL inhibitor. The metal-binding pharmacophore (MBP) fragment library makes it possible to systematically search for compounds targeting the binuclear Zn site of B1 MBL and identify the DPA framework. The DPA core framework for further optimization and SAR analysis found that 4-(3-aminophenyl) pyridine-2,6-dicarboxylic acid (25, Figure 10) had an IC50 value of 80 nM. Further experiments demonstrated that DPA showed a tendency of chelation with metal ions from NDM-1 to form a stable ternary complex of NDM-1:Zn(II):inhibitor, which acted as a metal-binding competitive inhibitor. When imipenem was used in combination with a DPA derivative, the resistance of Enterobacteriaceae producing NDM-1 to imipenem was reduced to a sensitive level [133].

Inspired by the DPA scaffold, Hinchliffe and coworkers designed and synthesized a series of 6-phosphonylmethylpyridine-2-carboxylic acid ester (PMPCs, 26, Figure 10) derivatives. According to bioelectronic isosteric theory, these derivatives substituted one of the carboxyl groups of DPA with a phosphonate. Without an isomerization step, the inhibitor competitively and slowly bound to NDM-1, with low IC50 values of 0.3–7.2 mM. SAR studies have indicated that both carboxylic and phosphonic acids are important pharmacophores for inhibiting NDM-1. When carboxylic or phosphonic acid exists alone, its inhibitory activity is greatly reduced. PMPCs inhibit NDM-1 by binding to the active site, not just by chelating metal ions. The efficacy, low toxicity, and affinity of PMPCs indicated that PMPCs and similar phosphonate compounds may be considered for the development of NDM-1 inhibitors [134].

##### Triazole-Thione Derivatives

**Figure 11:**
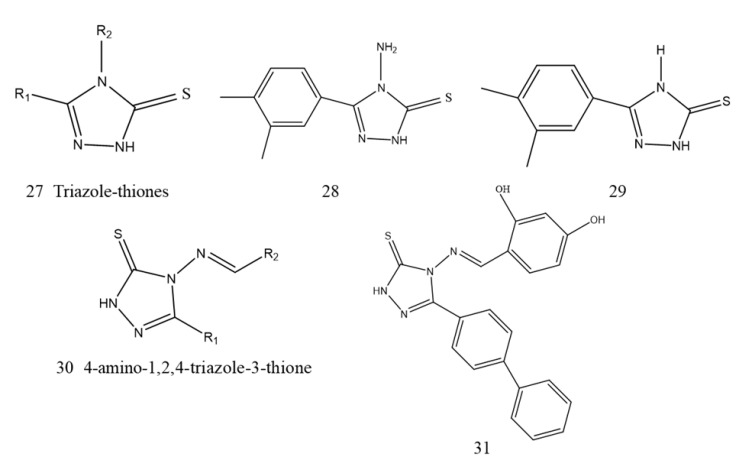
Triazole-thione derivatives as NDM-1 inhibitors.

Triazole thiones (27, Figure 11) are commonly reported as synthetic intermediates and have antibacterial, antiproliferative, and anti-inflammatory biological activity [135]. 1,2,4-Triazole-3-thione derivatives as metal ligands have been confirmed to inhibit clinically relevant MBLs, which are well adapted to target the catalytic site of di-zinc MBLs [136]. Based on the triazole-thione scaffold, fifty-four analogs of initial compounds 28 and 29 (28, 29, Figure 11) differing on their side chain at position 5 of the heterocycle were synthesized and evaluated, and identified. Nineteen inhibitors with an IC50 in the μM range toward at least one of the MBLs and five analogs inhibited at least four enzymes [137]. Recently, ninety 4-amino-1,2,4-triazole-3-thione-derived (30, Figure 10) bases were synthesized and characterized, and they changed at the 4th and 5th positions. The existence of the 4-position aryl moiety increased the potency by an average of 10 times. Several compounds are broad-spectrum inhibitors of MBLs, and compound 31 (31, Figure 11) was combined with colistin to restore the activity of colistin against NDM-1 clinical isolates [138].

#### 5.1.2. Boronic Acid Derivatives

**Figure 12:**
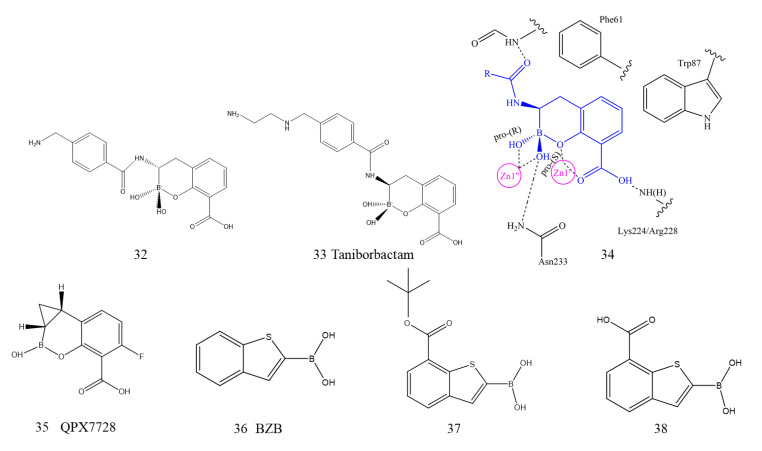
Boronic acid derivatives as NDM-1 inhibitors.

Since bortezomib was approved by the US FDA, borate compounds have been utilized as a new scaffold for β-lactamase inhibitor development. The boron atom in boronic acid is sp3 hybridized under physiological pH, and the geometric structure of the tetrahedron can fully simulate the instantaneous tetrahedral species formed in the catalytic process of hydrolytic enzymes [139]. Brem et al. reported that cyclic boronates, the first dual BL inhibitor, significantly inhibited both nucleophilic SBLs and zinc-dependent MBLs by imitating the common tetrahedral intermediate produced during the hydrolysis reaction. All five derivatives showed submicron/nanomolar inhibitor activity against NDM-1. It should be noted that with the combined use of compounds 32 and 33 (32, 33, Figure 12), the MIC of meropenem in all NDM-1-producing strains was reduced by up to 64 times. The X-ray crystallography structures of the B1 MBL complex with 34 (34, Figure 12) showed that first, the C-3 carboxylate oxygen interacts with both Zn2 and Lys224 (NDM-1 and BcII) or Arg228 (VIM-2). Second, the bicyclic phenyl-boronate ring of compound 34 on the active site of MBLs can interact hydrophobically with the conserved Trp87 and Phe61 residues. Finally, the two ‘exocyclic’ boron-binding hydroxyls coordinate with Zn1 and form hydrogen bonds with Asn233 and the NH of the acetylamino side chain. Cyclic boronates also potently inhibit the PBP targets of BLAs by the same mechanism of action [140].

The promotion effect of cyclic boron was further studied. By modifying the key structure of the bicyclic aromatic ring, methyl thioacetamide was found to be one of the most active imide substituents. By modifying the key structure, the compound QPX7728 (35, Figure 12) was ultimately obtained with a hydrophobic region composed of oleophilic side chains. QPX7728 displays ultrabroad-spectrum inhibition of SBL and MBL enzymes and is less influenced by modifications and efflux of the porin. In the complex crystal structures of QPX7728 with NDM-1, the catalytic water molecule is covalently bound to the boron atom of QPX7728. Due to the favorable bioavailability and biosafety of QPX7728, it is suitable for combined application with β-lactam antibiotics for the treatment of multidrug-resistant bacterial infections. The drug is currently in the late stage of preclinical development [141].

Starting from benzo-[b]-thiophene-2-boronic acid (BZB, 36 Figure 12) as an inhibitor of AmpC β-lactamase, Santucci et al. reported for the first time that a multiligand set of acyclic boronic acid of BZB analogs was able to inhibit clinically relevant BLs, including NDM-1. All the acyclic boronic acid analogs had low SBL micromolar inhibitor activity, while compounds 37 and 38 (37, 38, Figure 12) showed activity toward NDM-1 with IC50 values of 35.7 μM and 32.4 μM, respectively. Docking simulations suggested that the boronic group of derivatives coordinated two metal zinc ions, and the carboxyl groups interacted with key catalytic residues to form hydrogen bonds [142].

Both cyclic and acyclic boronic acids showed favorable NDM-1 inhibitory activity; however, the activity of the former was significantly stronger than that of the latter due to the double-ring structure of cyclic boric acid. Among them, compound 33 (taniborbactam, VNRX5133) with a dual-ring structure has been approved by the FDA to enter phase III clinical trials [143]. Taniborbactam is also a dual BLs inhibitor. The crystallographic results emphasize the ability of bicyclic borate to inhibit SBLs and MBLs by combining with tetrahedral (sp3) boron. The results further support boronic acid as a scaffold to design broad-spectrum BL inhibitors.

#### 5.1.3. Metal Chelating Inhibitors

**Figure 13:**
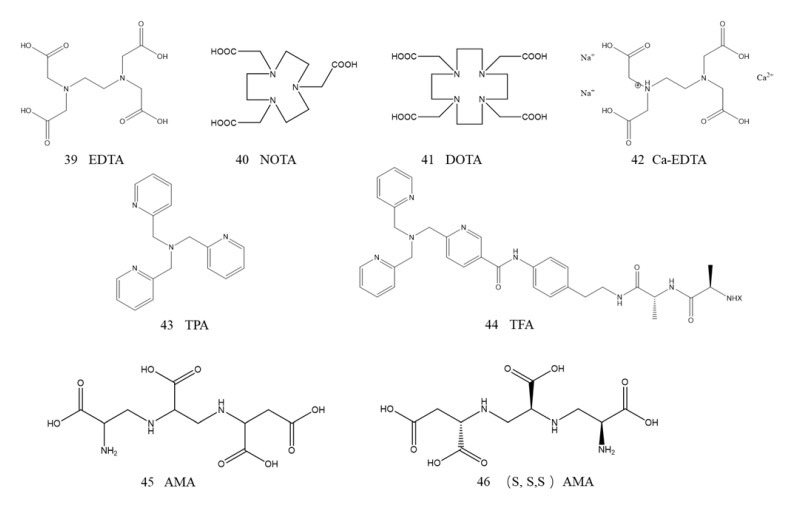
Metal chelating as NDM-1 inhibitors.

The main element of zinc-dependent enzyme inactivation is zinc deprivation, so it has been widely reported that metal complexing agents show MBL enzyme inhibition activity [144]. For example, ethylenediaminetetraacetic acid (EDTA, 39, Figure 13) is an MBL inhibitor with the function of sequestering and extracting key zinc in the active site, thus protecting the coadministered antibiotic from hydrolysis. Studies have shown that NDM-1 is more sensitive to EDTA than other MBLs, with an IC50 of 0.4 μM, indicating that EDTA possesses a strong ability to bind the Zn ions of NDM-1 [145]. Other metal chelators, 1,4,7-triazacyclononane-1,4,7-triacetic acid (NOTA, 40, Figure 13) and 1,4,7,10-tetra-azacyclononane-1,4,7,10-tetraacetic acid (DOTA, 41, Figure 13), demonstrated activity against NDM-1-producing bacteria. NOTA was more effective than DOTA in restoring antibiotic susceptibility [146]. Despite these promising effects, the toxicity of these nonselective chelating agents prevents them from being widely used in the clinic because they can chelate various metal ions, such as Zn, Fe, and Cu. Zn is the key element of essential enzymes, and excessive consumption of Zn by metal chelating agents may lead to disease [147]. An important exception was disodium calcium salt (Ca-EDTA, 42, Figure 12). It was reported that Ca-EDTA greatly reduced toxicity and was approved as an injection in Japan for the treatment of lead poisoning. Ca-EDTA significantly reduced the MICs of carbapenem antibiotics against all NDM-1-overexpressing bacteria. In a mouse model of sepsis, Ca-EDTA combined with imipenem and CILAStatin sodium treatment further reduced the bacterial load compared with imipenem or CILAStatin sodium treatments alone [105].

Schnaars et al. synthesized several novel selective zinc chelators based on a tris-picolylamine (TPA, 43, Figure 13) scaffold as putative MBL inhibitors. All new derivatives reduced the MIC of meropenem against MBL-harboring carbapenem-resistant strains and reduced HepG2 toxicity. Among them, one of the most promising compounds (TFA, 44, Figure 13) reduced the MIC of meropenem in MBL-expressing isolates and showed no activity on carbapenem serine-expressing strains, which indirectly demonstrated that Zn chelation of TPA had occurred.

King and coworkers established a cell-based NDM-1 inhibitor screening model by using natural product extracts from environmental microorganisms [104]. NDM-1 inhibitors were screened by the model strain [*E. coli* BW25113Δ bamBΔ tolCΔ araDAB: pLac(blaNDM-1)] in combination with a sublethal concentration of meropenem. Aspergillomarasmine A (AMA, 45, Figure 13) was identified from approximately 500 natural product extracts. It is a natural product that was isolated from the extract of Aspergillus versicolor fungus, which was an inhibitor of ACE and endothelin converting enzyme [148,149]. AMA had a highly selective inhibitory effect on NDM-1, with an IC50 of 4.1 ± 1.0 μM and Ki of 11 μM. The activity of meropenem on bacteria overexpressing NDM-1 was completely restored, but it was completely ineffective on SBLs. In contrast to the metal complexing agents mentioned above, AMA can also maintain efficacy in vivo. The survival study of mice showed that the bacterial load of NDM-1-positive *K. pneumoniae* in tissues was not affected by treatment with AMA and meropenem alone, while the combined treatment of AMA (10 mg·kg^−1^) and meropenem (10 mg·kg^−1^) significantly reduced the bacterial load in the spleen, and the 5-day survival rates were increased to 95%. Surprisingly, compared with the 50% lethal dose (LD50) of EDTA of 29 mg·kg^−1^, AMA exhibited low toxicity with an LD50 of 159.8 mg·kg^−1^. The inhibition of NDM-1 by AMA is irreversible, but the activity of the enzyme could be restored by adding excessive ZnSO4, which is consistent with a metal depletion mechanism. AMA represents a nontoxic candidate for an antibiotic adjuvant, and its high hydrophilicity with a CLog *P* value of −5 may limit further development and application in the clinic [104].

Soon after, Wright and coworkers completed the stereoselective total synthesis of AMA molecules, reassigned the absolute configuration of the three stereocenters to (S, S, S), and corrected the configuration (R, R, S) proposed by King for the first time. (S, S, S)-AMA (46, Figure 13) had the same inhibitory activity against NDM-1 as the first natural extract [150]. Seven new AMA derivatives were efficiently synthesized by different strategies. SAR studies clearly showed that the aspartic acid part of AMA and the carboxyl groups in C1, C4, C6, and C9 play a key role in NDM-1 inhibition. The combination of AMA derivatives and meropenem had a synergistic effect on the drug resistance of NDM-1-expressing *K. pneumoniae* and other gram-negative bacteria [151].

### 5.2. Covalently Bound Inhibitors

**Figure 14:**
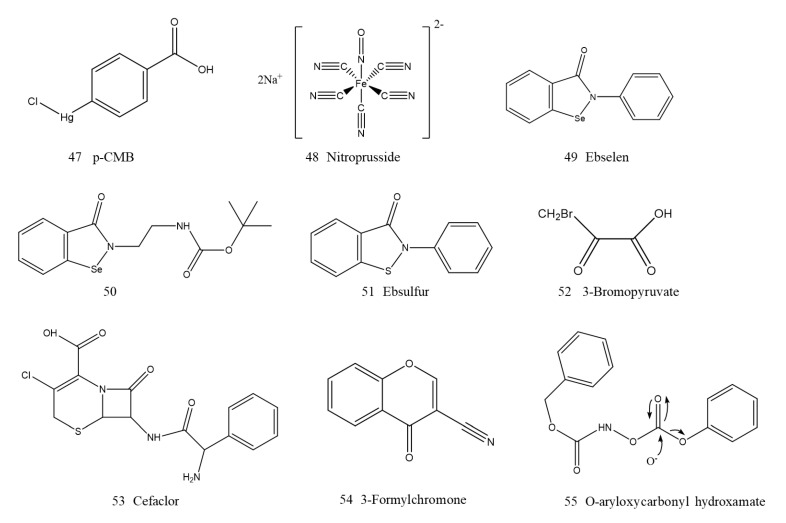
Covalently bound inhibitors of NDM-1.

NDM-1 has a Cys208 residue, which is an essential residue for the coordination with zinc ions to maintain a conserved active site structure, so this residue can potentially be a target in the design of NDM-1 inhibitors. Thomas et al. authenticated two irreversible thiol-modifying p-chloromercuribenzoate acids (p-CMB, 47, Figure 14) and nitroprusside (48, Figure 14) as NDM-1 inhibitors through high-throughput screening, which can be irreversibly covalently bound to the Cys208 residue on NDM-1 and exhibit inhibitory activity with IC50 values of 2.3 and 9.0 μM, respectively. Almost all enzyme activity was maintained despite the C208D mutation, and it was completely resistant to inhibitors. This acquirable resistance mutation demonstrated that covalent targeting of the conserved active-site Cys residue may have drawbacks as an NDM-1 inhibitor design strategy [152].

Ebselen (2-phenyl-1,2-benzoselenazol-3-one, 49, Figure 14) is a compound containing selenium used for the treatment of cerebral ischemia and stroke. It has also been shown to be a promising NDM-1 inhibitor by a cell-based screening method [153]. Enzymatic kinetic studies and ESI-MS analyses showed that ebselen could form a covalent S–Se bond with the Cys208 residue to binding to NDM-1, resulting in the removal of Zn2 from the active site, demonstrating a new inhibition mechanism and broad-spectrum inhibitory potential. In vitro, ebselen coadministration with meropenem resulted in 128-fold reductions in the MICs, thus restoring the activity of meropenem on NDM-1-positive *E. coli* [154]. A total of forty-six 1,2-benzisoselenazol-3(2H)-one scaffold derivatives were treated with ebselen, and many compounds displayed stronger synergistic activity with meropenem and better physiochemical properties than those of ebselen. Among them, compound 50 (50, Figure 14) could covalently bind to NDM-1 and transfer one zinc ion from the active site, showing strong synergistic activity with meropenem against clinical NDM-1 CRE isolates [155].

Su et al. further reported a potent covalent scaffold ebsulfur (51, Figure 14). Eighteen ebsulfur derivatives targeted NDM-1 with IC50 values ranging from 0.16 to 9 μM and effectively reversed the antibacterial activity of cefazolin against *E. coli* expressing NDM-1. Inhibition and equilibrium dialysis studies showed that there was a covalent and time-dependent relationship between ebsulfur and NDM-1 [156].

3-Bromopyruvate (52, Figure 14) is an active reactive electrophilic derivative of pyruvate, a cell metabolite that is clinically used for cancer treatment [157]. It has also been demonstrated to exhibit potential inhibitory activity on B1 and B2 MBLs, especially on NDM-1, with an IC50 of 2.57 μM. In addition, among the three clinical isolates that were NDM-1 positive, 3-bromopyruvate effectively restored the activity and reduced the MIC of five β-lactams antibiotics, such as cefotaxime and meropenem. A study on the inhibition mechanism suggested that 3-bromopyruvate may reversibly inhibit NDM-1 by covalently binding electrophilic methylene with Cys208 at the active center of NDM-1 [158].

Along this line of research, the Lys211 residue was also considered to be a promising “handhold” for the development of NDM-1 covalent inhibitors. Thomas et al. reported that the β-lactam drug cefaclor (53, Figure 14) is a time- and concentration-dependent covalent irreversible inactivator of NDM-1 with millimolar affinity. Cefaclor inactivation is mediated by a variety of pathways, including mediation by Lys211. Surprisingly, cefaclor was also demonstrated to be more effective as the substrate of NDM-1 than as an inactivator. Unfortunately, supratherapeutic doses of cefaclor are required to achieve NDM-1 inactivation in vitro, which hinders its clinical use against NDM-1 [159]. 3-Formylchromone (54, Figure 14) was identified as a novel covalent inhibitor of clinically relevant MBLs. The results of ESI-MS and single-site directed mutagenesis showed that cefaclor and Lys221 formed a covalent bond at the active site of NDM-1, while Lys211 is highly conserved and adjacent to the metal cluster of NDM-1 [160]. Recently, Thomas et al. also reported O-aryl oxycarbonyl hydroxamate (55, Figure 14) as a classical affinity label for NDM-1, which was bound with Lys211 in the substrate-binding site of NDM-1 [161].

### 5.3. Inhibitors with Other Mechanisms

In contrast to the screening and design philosophy of other NDM-1 inhibitors, Sully et al. constructed a peptide-conjugated phosphorodiamidate morpholino oligomer (PPMO) targeting NDM-1 mRNA and intervened in the expression of NDM-1 at the gene level. PPMO restored the sensitivity of NDM-1-positive strains to carbapenems in vitro. In a murine sepsis model infected with *E. coli* expressing blaNDM-1, PPMO in combination with meropenem significantly improved the survival rate, reduced the systemic bacterial burden, and alleviated inflammation. PPMO is a gene-specific therapeutic targeted to NDM-1, and this new strategy can rapidly design, synthesize and test sequence specificity against bacterial gene targets [162].

Chandar et al. screened ethanol extracts against the NDM-1 *E. coli* strain from the leaves of 240 medicinal plant species. Then, extracts from six plants, including *Hibiscus acetosella*, *Punica granatum*, and *Combretum albidum*, showed inhibitory activity on NDM-1 with an IC50 value ranging from 0.50 to 1.2 ng/µL, and the MIC was between 2.56 and 5.12 mg/mL. The mechanism of plant extracts inhibits NDM-1 enzyme activity by destroying the integrity of bacterial cell walls. When used in combination with antibiotics with FICI values of 0.09–0.375, all the plant extracts showed synergistic effects, which indicated the possibility of combined treatment with NDM-1 bacteria [163].

**Figure 15:**
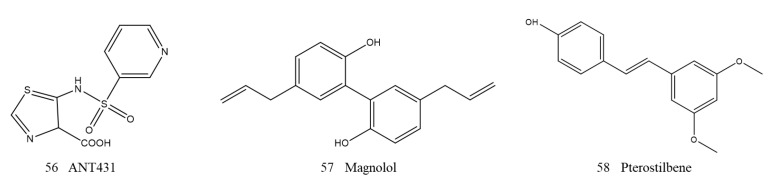
Other mechanisms inhibitors of NDM-1.

ANT431 (56, Figure 15), a specific competitive inhibitor of MBLs, is the result of design modifications based on the lead compound pyridine-2-carboxylic acid. ANT431 competitively inhibited the activity of NDM-1 and VIM-2 with Ki values of 0.29 and 0.195 μM, respectively. When the concentration of ANT431 was 30 μg/mL, it remarkably improved the activity of meropenem on recombinant NDM-1-positive engineered bacteria and reduced meropenem MICs to EUCAST breakpoint susceptibility levels in over 70% of highly resistant relevant clinical isolates. In a murine thigh infection model, meropenem in combination with ANT431 restored the efficacy of meropenem against *E. coli* NDM-1-producing bacteria. Compared with other metalloenzymes, such as ACE and GLY2, ANT431 displayed favorable selectivity and pharmacokinetic profile, which showed a high potential of patent medicine [164].

Liu et al. discovered two potent NDM-1 inhibitors that interact with amino acid residues. Magnolol (57, Figure 15), a natural product separated from the bark of magnolia trees, significantly inhibited the biological activity of NDM-1 with an IC50 value of 6.47 µg/mL and restored the effectiveness of meropenem in vitro against NDM-1-producing *E. coli*. The compound pterostilbene (58, Figure 15), which was originally isolated from red sandalwood, showed a synergistic inhibitory effect with meropenem against NDM-1-positive *E. coli*. The mechanistic analysis demonstrated that magnolol and pterostilbene were directly located in the catalytic pocket of NDM-1 and formed hydrogen bonds or hydrophobic interactions, thereby hindering substrate binding to NDM-1 and resulting in its inactivation. Furthermore, they inhibited the activity of NDM-1 without influencing the binding of NDM to Zn, which was different from the metal consumption mechanisms of other inhibitors [165,166].

Finally, novel synthetic peptide inhibitors were found and characterized by the surface localized antimicrobial display (SLAY), which enhanced the killing effect of carbapenem on NDM-1 *E. coli*. Approximately 1700 candidate peptide sequences were identified, among which only 37 peptides restored NDM-1-producing *E. coli* sensitivity to both meropenem and imipenem. Sequence analysis of 37 peptides showed that their specific amino acids were enriched, and each sequence encoded a residue with a positive charge. Conservative site-specific charge was helpful for peptides to penetrate the periplasm of bacteria and directly bind NDM-1 to inhibit enzymatic activity [167]. This approach provides a molecular platform for the discovery of NDM-1 inhibitors.

## 6. Conclusions

Over the past decade, NDM-1 and its variants have rapidly spread around the world. The gram-negative strain that produces NDM-1 poses a major public health threat because it can catalyze the hydrolysis of a variety of β-lactam antibiotics, including carbapenems, which is the last choice to treat infections caused by drug-resistant bacteria. Therefore, the active discovery of highly effective inhibitors and combination with β-lactam antibiotics has become an important strategy against NDM-1-expressing drug-resistant bacteria. Although a series of studies have been carried out on the structural characteristics, biological functions, and mechanism of action of NDM-1 in the past few years, none of the NDM-1 inhibitors have yet been approved for clinical application. The unclear catalytic mechanism of NDM-1 enzymes and various hydrolysis mechanisms of β-lactam antibiotics are crucial factors that hinder the development of NDM-1 inhibitors. The active site of NDM-1 gives the enzyme the characteristic that allows it to bind to a wide range of substrates, but the specific binding mode of NDM-1 to different substrates is different, which increases the difficulty in establishing the targeted active site inhibitor. Hopefully, with the development of computational biology, pharmaceutical chemistry, and other multidisciplinary knowledge and technology, the interaction mode of NDM-1 and substrate will be explained more accurately and thoroughly.

At present, compounds that can not only chelate zinc ions at the active site of NDM-1 but also form hydrogen bonds and/or salt bridges with amino acid residues at the binding site are the most promising inhibitors of NDM-1. Moreover, there are many metalloproteinases in the human body, and compounds targeting NDM-1 rather than human metalloproteinases may be prerequisites for the development of NDM-1 inhibitors in the future. At the same time, mutating NDM-1 tends to enhance the affinity of zinc ions in vivo and improve the tolerance to Zn deprivation, which poses a new challenge for the development of inhibitors in the future. In this paper, the overview of the superbug NDM-1 and the research progress of inhibitors provided are powerful references for the discovery and optimization of new inhibitors. Moreover, this review could provide MBL inhibitor-carbapenem combination strategies that complement the existing weaponry against CRE, with profound influence on human health.

## Figures and Tables

**Table 1:** Corresponding amino acid substitutions among NDM-1 and its variants and its first source of spread.

NDM-1 Variant	Location of Amino Acid(s) Substitution			Source Organism(s)
	A-Helices	β-Strands	Loop	
NDM-2	-	-	P28A	*A. baumannii*
NDM-3	D95N	-	-	*E. coli*
NDM-4	-	-	M154L	*E. coli*
NDM-5	-	V88L	M154L	*E. coli*
NDM-6	A233V	-	-	*E. coli*
NDM-7	D130N	-	M154L	*E. coli*
NDM-8	D130G	-	M154L	*E. coli*
NDM-9	-	-	E152K	*K. pneumoniae*
NDM-10	-	A74T	R32S, G36D, G69S, G200R	*K. pneumoniae*
NDM-11	-	-	M154V	*E. coli*
NDM-12	G222D	-	M154L	*E. coli*
NDM-13	D95N	-	M154L	*E. coli*
NDM-14	D130G	-	-	*A. lwoffii*
NDM-15	A233V	-	M154L	*E. coli*
NDM-16	A233V	V88L	M154L	*E. coli*
NDM-17	-	V88L	M154L, E170K	*E. coli*
NDM-18	-	-	QRFGD (44-48)	*P. rettgeri*
NDM-19	D130N	-	M154L, A233V	*E. coli*
NDM-20	-	V88L	M154L, R270H	*E. coli*
NDM-21	-	V88L	G69S, M154L	*E. coli*
NDM-22	-	-	M248L	*E. cloacae*
NDM-23	I101L	-	-	*K. pneumoniae*
NDM-24	-	V88L	-	*P. stuartii*
NDM-25	-	A55S	-	*K. pneumoniae*
NDM-26	G222S	V88L	M154L	*E. coli*
NDM-27	D95N, A233V	-	-	*E. coli*
NDM-28	A266V	-	-	*K. pneumoniae*
NDM-29	D130N	-	-	*K. pneumoniae*
NDM-30	D223Y	-	-	*K. oxytoca*
NDM-31	P171T	-	-	*C. werkmanii*
